# Differences in antibiotic susceptibility among gut-colonizing *Acinetobacter baumannii* isolated from hospital and community populations in Kenya

**DOI:** 10.11604/pamj.2025.52.111.47901

**Published:** 2025-11-13

**Authors:** Robert Maina Mugoh, Winnie Mutai, Victor Moses Musyoki, Charchil Ayodo, Beatrice Oduor, Moureen Jepleting, Teresa Ita, Susan Kiiru, Sylvia Omulo

**Affiliations:** 1 University of Nairobi, Department of Medical Microbiology and Immunology, Nairobi, Kenya,; 2 University of Nairobi Institute of Tropical and Infectious Diseases, Nairobi, Kenya,; 3 Washington State University Global Health-Kenya, Nairobi, Kenya,; 4 Centre for Microbiology Research, Kenya Medical Research Institute, Nairobi, Kenya,; 5 Paul G. Allen School for Global Health, Washington State University, Pullman, WA, USA

**Keywords:** *Acinetobacter baumannii*, gut colonization, antimicrobial resistance, community, hospital, Kenya

## Abstract

**Introduction:**

Acinetobacter baumannii is a critical priority multidrug-resistant bacterium. Colonization with resistant strains increases the risk of infection and facilitates transmission within healthcare and community settings. However, data on human colonization with drug-resistant A. baumannii remain limited, particularly in low- and middle-income countries. This study investigated and compared the antibiotic susceptibility profiles, resistance patterns, and genetic determinants of gut-colonising A. baumannii isolates from hospital and community settings across urban and rural areas in Kenya.

**Methods:**

a total of 125 A. baumannii isolates were analysed, comprising 86 community (54 urban, 32 rural) and 39 hospital (21 urban, 18 rural) isolates. Confirmatory identification and antimicrobial susceptibility testing were performed using VITEK®2 system. Conventional polymerase chain reaction (PCR) was employed to detect resistance genes, including bla_CTX-M_, bla_NDM1_, bla_OXA-23_, bla_OXA-48_, bla_SHV_, and bla_TEM_.

**Results:**

significant differences in antibiotic resistance were observed between hospital and community isolates (p≤0.003) across several antibiotics, including ampicillin/sulbactam, ciprofloxacin, ceftriaxone, cefepime, gentamicin, imipenem, meropenem, and sulfamethoxazole /trimethoprim. Rural community isolates showed near-universal susceptibility to most antibiotics tested, except for cefazolin and sulfamethoxazole/trimethoprim. All isolates were resistant to cefazolin but remained susceptible to tigecycline. No statistically significant differences in resistance were observed between urban and rural isolates within hospital or community settings (p > 0.05). Multidrug resistance was exclusively found in hospital isolates, 4 from rural, and 8 from urban, and among these, the bla_CTX-M_ gene was identified in five urban isolates, and bla_NDM1_ in two rural isolates.

**Conclusion:**

this study highlights the significant impact of the immediate environment (hospital vs. community) on A. baumannii antibiotic resistance profiles and underscores the need for tailored setting-specific interventions to address the evolving challenge of multidrug-resistant A. baumannii.

## Introduction

The increasing levels of antimicrobial resistance (AMR) pose a serious threat to global public health [1]. *Acinetobacter baumannii* is one of the ESKAPE pathogens, alongside *Enterococcus faecium, Staphylococcus aureus, Klebsiella pneumoniae, Pseudomonas aeruginosa*, and *Enterobacter* species. In 2017, the World Health Organization (WHO) designated *A. baumannii* as a critical priority pathogen due to its frequent association with multidrug resistance and its substantial role in healthcare-associated infections [2,3]. The organism´s ability to tolerate a range of temperatures and its capacity to persist on surfaces for extended periods contribute to its endemic presence in healthcare environments, where it can lead to various infections, including bacteremia, meningitis, urinary tract infections, and wound infections [4]. Although *A. baumannii* primarily occurs in healthcare settings, it is also found in community settings, particularly in tropical and subtropical regions. Individuals with underlying comorbidities such as diabetes, alcoholism, smoking, lung disease, and renal failure are at heightened risk of acquiring these infections [5,6].

Multidrug-resistant strains of *A. baumannii* exhibit resistance to multiple classes of antibiotics, including aminoglycosides, quinolones, penicillins, cephalosporins, and carbapenems [7]. The rising prevalence of carbapenem-resistant *A. baumannii* (CRAB) in recent years is concerning, as it severely restricts treatment options; carbapenems are typically reserved as a last-resort treatment for multidrug-resistant infections. The elevated rates of multidrug resistance (MDR) could be largely attributed to the presence of plasmid-encoded β-lactamases, reduced porin numbers and sizes that limit drug entry, and increased expression of efflux pumps [8].

The scarcity of effective drugs for treating *A. baumannii infections* presents challenges for healthcare facilities worldwide [9]. Additionally, many low- and middle-income countries lack the diagnostic capacities necessary to accurately isolate and identify *A. baumannii*, which hampers the implementation of appropriate treatment protocols, such as antibiotic susceptibility testing (AST) and antimicrobial stewardship programs [9,10].

Colonization with extended-spectrum β-lactamase (ESBL)-producing *A. baumannii* is often asymptomatic but increases the risk of multidrug-resistant infections and facilitates transmission to non-colonized individuals. Notably, even individuals without prior exposure to healthcare facilities or antibiotics, such as travelers returning from sub-Saharan Africa and South Asia, are colonized with ESBLs, underscoring the significant colonization pressure within communities [5,6,10].

This study aimed to assess the antibiotic susceptibility profiles of *A. baumannii* isolated from asymptomatic participants enrolled in the Antimicrobial Resistance in Communities and Hospitals (ARCH) study, conducted in selected hospitals and communities in Kenya between January 2019 and March 2020. We also investigated the presence of genes associated with resistance to cephalosporins and carbapenems. Despite the increased risk of colonization with multidrug-resistant organisms, there is limited information on *A. baumannii* colonization in hospitals and communities globally, particularly in Africa. The scarcity of published information underscores the continued public health relevance of these findings, five years later.

## Methods

**Study setting:** the study utilized archived stool and rectal samples from the ARCH study conducted between January 2019 and March 2020. The ARCH study enrolled participants from urban (Kibera) and rural (Asembo) settings. Mbagathi County Hospital, which serves Kibera and neighboring communities, was selected for hospital enrollment. In Asembo, inpatients from Siaya County Referral Hospital, Bondo Sub-County Hospital, and St. Elizabeth (Lwak) Hospital were enrolled. Our previous publication provides further insights into the study sites [11].

**Sample collection and processing:** samples were cultured on HardyCHROM™ Carbapenem Resistant Enterobacterales (CRE) agar plates and HardyCHROM™ Extended-spectrum beta-lactamase (ESBL) agar plates to selectively isolate presumptive *A. baumannii*. After incubating at 37°C for 18-24 hours, smooth off-white colonies, morphologically consistent with *A. baumannii*, were subcultured on TSA and further incubated for 18 - 24 hours at 37°C. Purified isolates were emulsified in 50% glycerol phosphate-buffered saline and archived at -80°C for subsequent analysis.

**Bacterial identification and AST:** archived isolates were revived by thawing at -20°C overnight and at 4°C for 1 hour before processing at room temperature (18-25°C). Isolates were cultured on TSA plates and incubated aerobically at 37°C for 18-24 hours. Bacterial suspensions were prepared from each archived isolate by emulsifying single colonies in 3mL of 0.45% saline and adjusting to a 0.5 McFarland standard using the DensiCHEK Plus (bioMérieux, France). The suspension was then loaded into the VITEK®2 automated system (bioMérieux, France), and isolate identity was confirmed using the Gram-negative ID VITEK®2 automated system cards (bioMérieux, France). Antibiotic susceptibility testing was conducted on *A. baumannii* isolates using the VITEK®2 AST-GN71 card (bioMérieux, France), which contained a panel of 11 antibiotics (Ampicillin/sulbactam; SAM≤ 4/2 μg/mL, Cefazolin CEZ≤ 4/2 μg/mL, Cefepime FEP ≤ 2 μg/mL, Ceftriaxone CRO≤ 1 μg/mL, Ciprofloxacin CIP≤ 0.5 μg/mL, Gentamicin GEN ≤ 4 μg/mL, Imipenem IMP≤ 1 μg/mL, Meropenem MEM ≤ 0.5 μg/mL, Sulfamethoxazole/trimethoprim SXT ≤ 1 μg/19 μg/mL, Tigecycline TGC ≤ 0.75μg/mL, Tobramycin TOB ≤ 8μg/mL). Minimum inhibitory concentration (MIC) values were interpreted according to the 2020 Clinical Laboratory Standards Institute guidelines [12]. Although *A. baumannii* is intrinsically resistant to cefazolin due to chromosomal mechanisms; this antibiotic was included in testing to identify any potential extended-spectrum β-lactamases (ESBLs) or other acquired resistance genes that could modify susceptibility.

**Detection of resistance genes:** *K. pneumoniae* ATCC 700603 was used as a positive control strain for both phenotypic and genotypic assays. Nuclease-free water was used as a negative control for molecular assays. Deoxyribonucleic acid (DNA) was extracted using the DNeasy Ultra Clean Kit (Qiagen, Maryland, USA), a bead-based method for the isolation of genomic DNA, following the manufacturer´s instructions. Briefly, bacterial cells were suspended in a bead solution-containing tube, and beads were added along with the lysis solution. On vortexing, microorganisms were lysed by a combination of heat, mechanical energy, and detergent. DNA from lysed cells is bonded to a silica spin filter, washed, and recovered in DNA-free Tris buffer. The extracted DNA was stored at -80°C until analyzed using conventional PCR. Table 1 shows the primers used to screen isolates for resistance genes encoding resistance to cephalosporins (*bla*_CTX-M_, *bla*_SHV_, and *bla*_TEM_) and carbapenems (*bla*_NDM1_, *bla*_OXA-23_, and *bla*_OXA-48_) [13] using conventional PCR.

**Table 1:** primers used to detect antimicrobial resistance genes in *Acinetobacter baumannii* isolates

Gene	Primer sequence	Size (bp)	Temp (°C)
†*bla*_CTX-M_	F-5'-ATGTGCAGYAACAGTAARRGTKATGGC-3'	593	60
	R-5'-TGGGTRAARTARGTSAACAGAAYCAGC GG-3'		
†*bla*_SHV_	F-5'-TTCGCCTGTGTATTATCTCCCTG-3'??	854	50
	R-5'-TTAGCGTTGCCAGTGYTCG-3'		
†*bla*_TEM_	F-5'-ATGAGTATTCAACATTTCCG-3'	867	50
	R-5'-CCAATGCTTAATCAGTGACG-3'		
††*bla*_NDM1_	F-5'-GAGATTGCCGAGCGACTTG-3'	813	61
	R-5'-CGAATGTCTGGCAGCACACTT-3'		
††*bla*_OXA23_	F-5'-CTTGCTATGTGGTTGCTTCTC-3'	650	50
	R-5'-ATCCATTGCCCAGTC-3'		
*bla_OXA48_	F-5'-TGTTTTTGGTGGCATCGAT-3'	177	60
	R-5'-GTAAMRATGCTTGGTTCGC-3'		

† Class A metallo-β-lactamases; ††Ambler Class B; *Class D carbapenemases. F, forward primer; R, reverse primer; bp: base pairs for amplicon size

A singleplex assay was conducted using 25 μL reaction mix containing 12 μL of PCR master-mix (Taq DNA polymerase supplied in a proprietary reaction buffer (pH 8.5), dATP 400 μM, dGTP 400 μM, dCTP 400 μM, dTTP 400 μM, MgCl2 23 mM) (Promega, Madison, USA) with pairs of specific primers (10 pmol) at 1μL each, both forward and reverse primers (Table 1), and 1μL DNA template and 12 μL nuclease-free water. PCR was conducted using the Gene AMP 9700 PCR system (Applied Biosystems, USA) in 0.2ml PCR tubes. Amplification was done by initial heating at 95°C for 5 minutes, followed by 30 cycles of denaturation at 95°C for 30 seconds, annealing at the specified primer temperature for 30 seconds, extension at 72°C for 2 minutes, with a final extension of 72°C for 7 minutes. Visualization of amplified PCR products was performed using a 1.5% agarose gel with SYBR Green dye, along with a 1kb molecular ladder. The gel was run for 60 minutes in 5X Tris-acetate Ethylenediaminetetraacetic Acid buffer at 90 volts, 65 mA, and 6 watts. Bands corresponding with respective sequence amplicon sizes (Table 1) were visualized under a UV transilluminator.

**Data analysis:** data from the VITEK®2 system were exported to MS Excel for cleaning and management. Isolates with resistance to 3^rd^ or 4^th^ generation cephalosporins with MIC values ≥ 32 μg/mL for cefepime, ≥ 64 μg/mL for ceftriaxone, and ≥ 32 μg/mL for ceftazidime were presumed to likely harbor ESBLs. Similarly, isolates resistant to carbapenems with MIC ≥ 8 μg/mL for ertapenem, ≥ 8 μg/mL for meropenem, or ≥ 8 μg/mL for imipenem were considered likely to carry carbapenemases. Isolates that were resistant to at least one antibiotic from three or more antibiotic classes were classified as multidrug-resistant [8]. Data were analyzed using SPSS version 26.0 (IBM Corp., NY, USA). Differences in AST results were compared using Fisher´s exact test, and the presence and distribution of genes encoding β-lactam resistance were evaluated across community and hospital isolates.

**Ethical considerations:** ethical approval was sought from the KNH-UON Ethics and Research Committee (ERC) and approved under Ref no. P504/06/2022. Archived isolates were obtained from stool samples from a study previously approved by KNH-UoN ERC Ref no. P164/03/2018. An ethics review committee waiver of informed consent was obtained.

## Results

A total of 138 presumptive *Acinetobacter* isolates were selectively revived and microbiologically confirmed, of which 125 viable isolates were identified as *A. baumannii*. These comprised 86 (69%) community isolates (54 urban and 32 rural) and 39 (31%) hospital isolates (21 urban and 18 rural) (Table 2).

**Table 2:** antibiotic susceptibility patterns of *Acinetobacter baumannii* isolates across the different study sites

Site	Antibiotic												
			CEZ	CIP	CRO	FEP	GEN	IMP	MEM	SAM	SXT	TGC	TOB
Hospital	AST result												
Rural (N = 18)	S	n (%)	0 (0)	16 (89)	3 (17)	14 (78)	14 (78)	16 (89)	16 (89)	15 (83)	5 (28)	18 (100)	14 (78)
	I	n (%)	0 (0)	0 (0)	10 (56)	1 (6)	1 (6)	0 (0)	0 (0)	0 (0)	0 (0)	0 (0)	3 (17)
	R	n (%)	18 (100)	2 (11)	5 (28)	3 (17)	3 (17)	2 (11)	2 (11)	3 (17)	13 (72)	0 (0)	1 (6)
Urban (N = 21)	S	n (%)	0 (0)	14 (67)	3 (14)	13 (62)	14 (67)	15 (71)	15 (71)	15 (71)	11 (52)	14 (67)	21 (100)
	I	n (%)	0 (0)	0 (0)	9 (43)	0 (0)	0 (0)	0 (0)	0 (0)	1 (5)	0 (0)	7 (33)	0 (0)
	R	n (%)	21 (100)	7 (33)	9 (43)	8 (38)	7 (33)	6 (29)	6 (29)	5 (24)	10 (48)	0 (0)	0 (0)
Community													
Rural (N = 32)	S	n (%)	0 (0)	32 (100)	6 (19)	31 (97)	32 (100)	32 (100)	32 (100)	32 (100)	19 (59)	32 (100)	32 (100)
	I	n (%)	0 (0)	0 (0)	26 (81)	1 (3)	0 (0)	0 (0)	0 (0)	0 (0)	1 (3)	0 (0)	0 (0)
	R	n (%)	32 (100)	0 (0)	0 (0)	0 (0)	0 (0)	0 (0)	0 (0)	0 (0)	12 (38)	0 (0)	0 (0)
Urban (N = 54)	S	n (%)	0 (0)	54 (100)	13 (24)	53 (98)	54 (100)	54 (100)	54 (100)	53 (98)	40 (74)	54 (100)	52 (96)
	I	n (%)	2 (4)	0 (0)	40 (74)	1 (2)	0 (0)	0 (0)	0 (0)	0 (0)	0 (0)	0 (0)	1 (2)
	R	n (%)	52 (96)	0 (0)	1 (2)	0 (0)	0 (0)	0 (0)	0 (0)	1 (2)	14 (26)	0 (0)	1 (2)

CEZ; cefazolin, CIP; ciprofloxacin, CRO; ceftriaxone, FEP; cefepime, GEN; gentamicin, IMP; imipenem, MEM; meropenem, SAM; ampicillin/sulbactam, SXT; sulfamethoxazole/trimethoprim, TGC; tigecycline, TOB; tobramycin. AST result: S, susceptible; I, intermediate resistance; R, resistant

**Antimicrobial susceptibility:** all *A. baumannii isolates*, regardless of setting, were resistant to cefazolin but susceptible to tigecycline. Significant differences in antibiotic resistance were observed between community and hospital isolates. Resistance was higher in hospital isolates for ampicillin/sulbactam, ciprofloxacin, ceftriaxone, cefepime, gentamicin, imipenem, and meropenem (all p < 0.001), as well as sulfamethoxazole/trimethoprim (p = 0.003; Table 3). No statistically significant differences in resistance were observed between urban and rural hospital isolates, or urban and rural community isolates (all p > 0.05). Except for cefazolin and sulfamethoxazole/trimethoprim, all isolates from rural community participants were susceptible to all other antibiotics tested. Four (22%) rural hospital isolates, and 8 (38%) urban hospital isolates were classified as multidrug-resistant; no community isolate showed multidrug-resistant phenotypes. Six MDR profiles were identified, with CEZ-CIP-CRO-FEP-GEN-IMP-MEM-SAM-SXT being the only shared profile, observed in 5 (63%) urban isolates and 1 (25%) rural isolate. Other profiles occurred less frequently and were restricted to either urban or rural hospital isolates (Table 4).

**Table 3:** antibiotic susceptibility among *Acinetobacter baumannii* isolates from hospital and community sites

	Resistant isolates (%)								
	CIP	CRO	FEP	GEN	IMP	MEM	SAM	SXT	TOB
Hospital									
Rural (N=18)	2 (11)	5 (28)	3 (17)	3 (17)	2 (11)	2 (11)	3 (17)	13 (72)	1 (6)
Urban (N=21)	7 (33)	9 (43)	8 (38)	7 (33)	6 (29)	6 (29)	5 (24)	10 (48)	0 (0)
Community									
Rural (N=32)	0 (0)	0 (0)	0 (0)	0 (0)	0 (0)	0 (0)	0 (0)	12 (38)	0 (0)
Urban (N=54)	0 (0)	1 (2)	0 (0)	0 (0)	0 (0)	0 (0)	1 (2)	14 (26)	1 (2)

CIP; ciprofloxacin, CRO; ceftriaxone, FEP; cefepime, GEN; gentamicin, IMP; imipenem, MEM; meropenem, SAM; ampicillin/sulbactam, SXT; sulfamethoxazole/trimethoprim, TOB; tobramycin

**Table 4:** distribution of multidrug resistance phenotypes among *Acinetobacter baumannii* hospital isolates

	Resistant isolates (%)		
MDR phenotype	Rural (n=4)	Urban (n=8)	Total (N=12)
CEZ-CIP-CRO-FEP-GEN-IMP-MEM-SAM-SXT	1 (25)	5 (63)	6 (50)
CEZ-CIP-CRO-FEP-GEN-IMP-MEM-SAM-SXT-TOB	1 (25)	0 (0)	1 (8)
CEZ-CIP-CRO-FEP-GEN-SXT	0 (0)	2 (25)	2 (16)
CEZ-CRO-FEP-IMP-MEM-SXT	0 (0)	1 (13)	1 (8)
CEZ-CRO-FEP-SAM-SXT	1 (25)	0 (0)	1 (8)
CEZ-CRO-GEN-SXT	1 (25)	0 (0)	1 (8)

CEZ; cefazolin, CIP; ciprofloxacin, CRO; ceftriaxone; FEP; cefepime, GEN; gentamicin, IMP; imipenem, MEM; meropenem, SAM; ampicillin/sulbactam, SXT; sulfamethoxazole/trimethoprim, TOB, Tobramycin

Among the 12 phenotypic MDR isolates, seven carried at least one antimicrobial resistance gene detected by PCR, while the remaining five did not show any of the screened resistance genes. Of the six targeted genes associated with beta-lactam and carbapenem resistance (*bla*_CTX-M_, *bla*_NDM1_, *bla*_OXA-23_, *bla*_OXA-48_, *bla*_SHV_ and *bla*_TEM_), only two were detected: *bla*_CTX-M_ in five urban isolates and *bla*_NDM1_ in two rural isolates.

## Discussion

Antimicrobial resistance remains a major global public health threat, with colonization by drug-resistant bacteria increasing the risk of infection and transmission in healthcare and community settings. This study aimed to investigate and compare the antibiotic susceptibility profiles, resistance patterns, and genetic determinants of gut-colonising *A. baumannii* isolates from hospital and community settings across urban and rural areas in Kenya. The findings highlight significant disparities in antibiotic resistance between hospital and community *A. baumannii* isolates, with hospital isolates showing substantially higher resistance rates to multiple clinically important antibiotics such as ampicillin-sulbactam, ciprofloxacin, ceftriaxone, cefepime, gentamicin, imipenem, meropenem, and sulfamethoxazole-trimethoprim. These findings align with observations from other studies that reported high resistance profiles in hospital settings [14,15], suggesting that selective pressure from antibiotic use within hospitals may play a significant role in shaping these resistance patterns.

In contrast, rural community isolates had low resistance levels, exhibiting susceptibility to nearly all antibiotics tested, except for cefazolin and sulfamethoxazole/trimethoprim. Similar observations have been reported in veterinary medicine [16] and may reflect a relatively stable environmental reservoir of *A. baumannii in* rural settings, where the selective pressure from broad-spectrum antibiotic use is minimal [7]. This “baseline susceptibility” in these communities may provide insights into the evolutionary trajectory of resistance, highlighting the impact of human activity in urban and hospital environments. Furthermore, universal resistance to cefazolin across all isolates, regardless of origin, suggests its intrinsic resistance to this antibiotic, a known characteristic of *A. baumannii* strains [17]. Conversely, the universal susceptibility to tigecycline highlights its potential as a last-line treatment option against *A. baumannii* in these settings.

The exclusive presence of multidrug-resistant phenotypes in hospital isolates in this study raises concerns regarding the possibility of diminishing treatment options over time. The identification of six distinct MDR phenotype profiles among hospital isolates, with the CEZ-CIP-CRO-FEP-GEN-IMP-MEM-SAM-SXT profile being the only shared phenotype between urban and rural hospitals, highlights a complex evolution of resistance within these environments. Additionally, since no significant differences in resistance were observed between urban and rural isolates within either the hospital or community settings, we surmise that the immediate environment, specifically high antibiotic use in hospitals versus presumably lower use in the community, exerts a stronger selective pressure than the broader geographical context [11]. These findings indicate that the micro-environment may be the more critical unit of analysis, and that infection control and antibiotic stewardship interventions may need to be tailored at a more specific, hospital-level rather than relying on broader regional strategies, as has been suggested by other studies involving this bacterium [15].

The detection of specific resistance genes, such as *bla*_CTX-M_ in five urban isolates and *bla*_NDM1_ in two rural isolates, provides important insights into the molecular mechanisms underlying resistance to beta-lactam and carbapenem antibiotics. In this study, the reference strain used as a positive control amplified for the specific ESBL-associated gene but not carbapenemase genes, consistent with its known genotype. Notably, the absence of other targeted resistance genes in the hospital and community isolates, particularly *bla_OXA_* genes, which have been reported globally as the predominant mechanism of carbapenem resistance in *A. baumannii* raises questions about the genetic determinants driving resistance in these populations. The observed partial overlap between the phenotypic MDR and genotypic detection of resistance genes reported in this study may be explained by alternative resistance mechanisms, such as efflux pump overexpression, porin loss or hyperproduction of AmpC β-lactamases [18]. This observation aligns with findings from previous studies conducted in Kenya that demonstrated the emergence and persistence of MDR *A. baumannii* in hospital settings. Studies by Huber *et al*. [19] and Revathi *et al*. [20] reported the presence of *bla_NDM__-__1_* and *bla_OXA-23_* producing *A. baumannii* in hospital settings in Kenya, indicating the early dissemination of carbapenem-resistant strains in the region. Similarly, investigations by Musyoki *et al*. [21] and Musila *et al*. [22] documented widespread resistance across multiple antibiotic classes and circulation of carbapenemase genes, including *bla_OXA-23_, bla_NDM__-__1__,_* and *bla_OXA-58_*, among high-risk sequence types such as ST1, ST2, and ST1475.

**Limitations:** this study had several limitations. First, the sample selection was limited to a few sites, and the isolates selectively represented a resistant subpopulation and not the total colonization burden, which limits the generalizability of the findings. Additionally, the study primarily focused on specific resistance genes, potentially overlooking other mechanisms contributing to antibiotic resistance. Future investigations could incorporate whole-genome sequencing and broader genetic analyses to identify and characterize these mechanisms. Lastly, a longitudinal approach would guide assessment changes in resistance over time, providing insights into the evolving dynamics of *A. baumannii* resistance.

## Conclusion

This study underscores the need for robust infection control and antibiotic stewardship measures in hospitals to address multidrug-resistant *A. baumannii*. While isolates from hospitals had high levels of resistance, rural community isolates had near-universal susceptibility, indicating a less affected reservoir. Further, the distinct multidrug-resistant phenotypes in urban and rural hospitals highlight the significance of local factors over geographical distinctions. Our limited identification of resistance genes implies that other non-targeted mechanisms play a role in resistance transmission in these settings. Future strategies should prioritize tailored interventions based on specific local resistance patterns.

### What is known about this topic

- Antimicrobial resistance is a critical global health threat in this century due to an increased resistance to different pathogens;
- Acinetobacter baumannii is one of the critical-priority pathogens on the World Health Organization priority list of antibiotic-resistant bacteria for effective drug development.

### What this study adds

- Colonizing A. baumannii can proliferate and develop antimicrobial resistance in different settings; these variations may be attributed to high patient populations, access to antibiotics, and prescription practices in urban hospital settings that have demonstrated higher risks and exposure to the bacteria;
- The occurrence of MDR A. baumannii phenotypes is an issue in hospitals, as isolates from community settings showed resistance only to two drug classes; therefore, hospitals, as aforementioned, harbor the highest risks for the development and spread of drug resistance.
- Two of the genes were found in seven of the 15 identified MDR strains of A. baumannii; the bla_CTX-M_ gene was the predominant resistant gene responsible for the development of resistance to beta-lactams like cephalosporins. However, the frequency of this gene in hospital settings is disproportionately higher in urban hospitals, positioning them as key reservoirs for ESBL-producing microbes.
